# Supplementary material for: Unbiased profiling of CRISPR RNA-guided transposition products by long-read sequencing
Source: Mob DNA. 2021 Jun 8;12:13. doi: 10.1186/s13100-021-00242-2 (PMC8188705; doi:10.1186/s13100-021-00242-2)
Supplement: Supplementary file 1 — Additional file 1: Supplementary Figure 1. SMRT-seq approach to profile genome-wide transposition products. a, Representative read coverage from whole-genome SMRT-sequencing of E. coli strain BW25113, transformed with vectors encoding the WT V. cholerae CRISPR-transposon system. Read coverage was 90-120X coverage across the full-length genome. b, Representative CCS reads from whole-genome SMRT-seq data in a, aligned to the parental strain reference genome at the target site (maroon triangle). Purple blocks indicate large insertions corresponding to genomic RNA-guided DNA integration events. Supplementary Figure 2. Genome-wide analyses of integration events extracted from SMRT-seq data. a, Integration events were determined from SMRT-seq CCS reads containing transposons flanked by genome-mapping sequences, for both simple insertion and cointegrate transposition products. Data are shown for the S. hofmannii CRISPR-Tn using either sgRNA-252 (left) or sgRNA-261 (right); target sites are denoted by maroon triangles. Note the scaling on the y-axis. b, Data for the V. cholerae CRISPR-Tn encoding WT (left) or D90A-TnsA (right), shown as in a. c, Data for the V. cholerae CRISPR-Tn encoding WT (left) or D90A-TnsAB fusion (right), shown as in a. [file 13100_2021_242_MOESM1_ESM.docx]

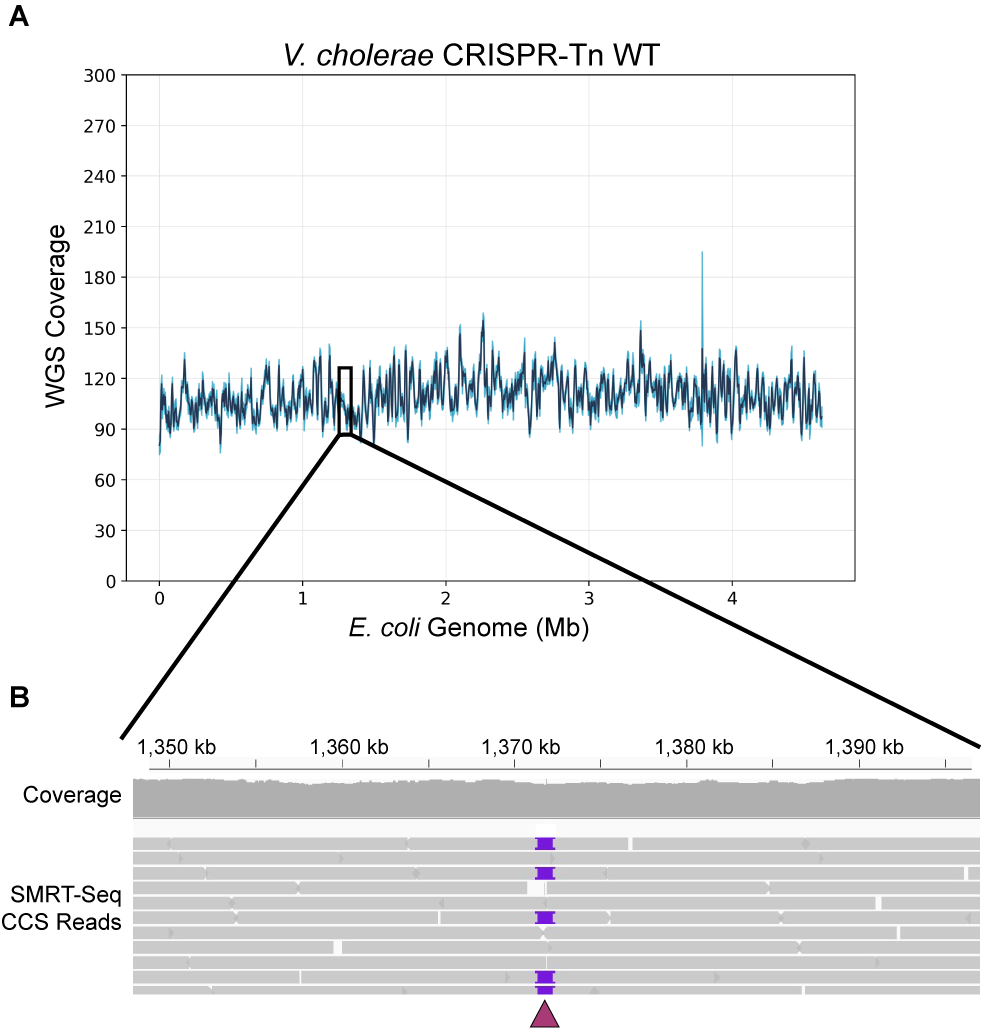


**Supplementary Figure 1.** SMRT-seq approach to profile genome-wide transposition products. **a,** Representative read coverage from whole-genome SMRT-sequencing of *E. coli* strain BW25113, transformed with vectors encoding the WT *V, cholerae* CRISPR-transposon system. Read coverage was 90-120X coverage across the full-length genome. **b,** Representative CCS reads from whole-genome SMRT-seq data in **a**, aligned to the parental strain reference genome at the target site (maroon triangle). Purple blocks indicate large insertions corresponding to genomic RNA-guided DNA integration events.

**
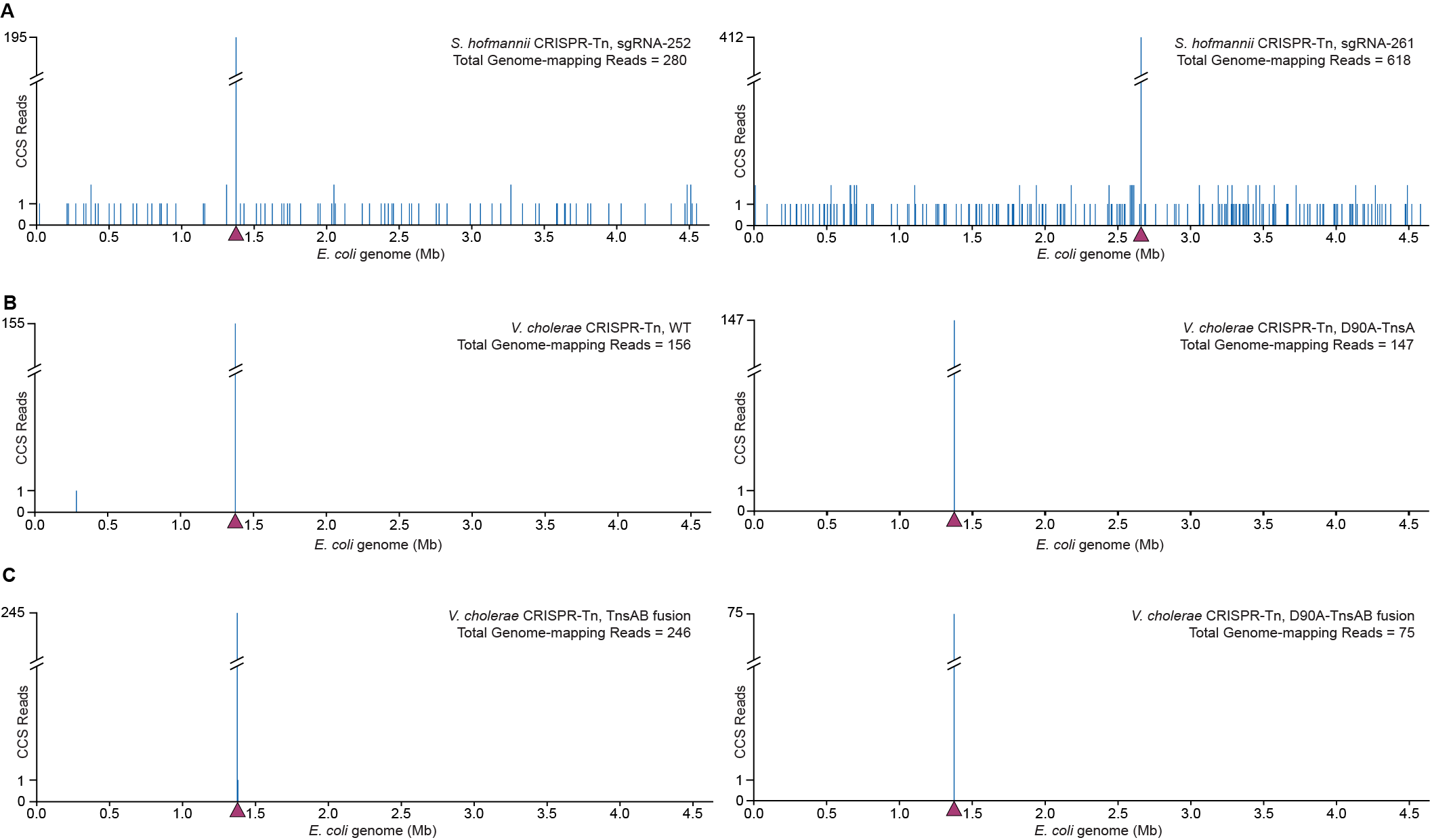
**

**Supplementary Figure 2.** Genome-wide analyses of integration events extracted from SMRT-seq data. **a,** Integration events were determined from SMRT-seq CCS reads containing transposons flanked by genome-mapping sequences, for both simple insertion and cointegrate transposition products. Data are shown for the *S. hofmannii* CRISPR-Tn using either sgRNA-252 (left) or sgRNA-261 (right); target sites are denoted by maroon triangles. Note the scaling on the y-axis. **b,** Data for the *V. cholerae* CRISPR-Tn encoding WT (left) or D90A-TnsA (right), shown as in **a**. **c,** Data for the *V. cholerae* CRISPR-Tn encoding WT (left) or D90A-TnsAB fusion (right), shown as in **a**.
